# Incorporating graph representation and mutual attention mechanism for MiRNA-MRNA interaction prediction

**DOI:** 10.3389/fgene.2025.1637427

**Published:** 2025-07-17

**Authors:** Tai-Long Shi, Lei Wang, Leon Wong, Zhu-Hong You, Chang-Qing Yu, Chen Jiang, Si-Zhe Liang

**Affiliations:** ^1^ School of Electronic Information, Xijing University, Xi’an, China; ^2^ Guangxi Key Lab of Human-Machine Interaction and Intelligent Decision, Guangxi Academy of Sciences, Nanning, China; ^3^ School of Computer Science and Technology, China University of Mining and Technology, Xuzhou, China; ^4^ College of Big Data and Internet, Shenzhen Technology University, Shenzhen, China; ^5^ School of Computer Science, Northwestern Polytechnical University, Xi’an, China

**Keywords:** miRNA-Target mRNA interactions, mutual attention mechanisms, BiLSTM, fastText, GraRep

## Abstract

**Introduction:**

Predicting interactions between microRNAs (miRNAs) and messenger RNAs (mRNAs) is crucial for understanding gene expression regulation mechanisms and their roles in diseases. Existing prediction methods face significant limitations in simultaneously handling RNA sequence complexity and graph structural information.

**Methods:**

We propose GRMMI, a framework that effectively leverages both sequence and node features by combining FastText-pretrained sequence embeddings with GraRep graph embeddings to capture semantic and topological information. The method introduces antisense-aware sequence processing that reverses mRNA orientation to better simulate the natural miRNA-mRNA complementary binding mechanism. Additionally, GRMMI employs cross-sequence mutual attention architecture that enables deep exploration of inter-RNA dependencies beyond traditional single-sequence analysis limitations. Unlike existing approaches that rely primarily on sequence-based features, GRMMI achieves multi-dimensional information fusion by integrating CNN-BiLSTM architecture with mutual attention mechanisms.

**Results:**

Evaluation on the MTIS-9214 dataset shows that GRMMI achieves an AUC of 0.9347 and accuracy of 86.65%.

**Discussion:**

Case studies confirm the practical utility of GRMMI in identifying biologically significant RNA interactions, providing valuable insights for disease mechanism research and therapeutic target discovery.

## Highlights

• This study introduces a mutual attention mechanism, enabling the model to effectively capture the complex interaction features between miRNAs and mRNAs, thereby uncovering additional latent associative information.
• The mRNA sequence is inputted in reverse order into the model, taking into account the biological characteristics of miRNA and mRNA binding.
• An improved FastText method is used as the pre-training model for RNA sequences, allowing for the generation of feature embeddings more aligned with the experimental objectives during deep mining.

## Introduction

Non-coding RNAs (ncRNAs) are RNA molecules that regulate gene expression at the post-transcriptional level and lack protein-coding potential. They interact with other biomolecules as functional macromolecules to modulate various cellular processes. With the growing body of research on the ncRNAs, it has been established that both microRNAs, referred to as miRNAs, and messenger RNAs referred to as mRNAs are central to numerous biological processes. MiRNAs, approximately 22 nucleotides in length, were first discovered in *Caenorhabditis elegans* in 1993. They regulate gene expression post-transcriptionally in plants and animals by binding to specific mRNA sequences through complementary base pairing, influencing translation efficiency or promoting mRNA degradation, ultimately resulting in the suppression of protein synthesis (Kim and Croce, 2023; Walgrave et al., 2021).

MiRNA genes are transcribed into pri-miRNAs in the nucleus, processed by the Drosha enzyme to form pre-miRNAs, and then transported to the cytoplasm, where the Dicer enzyme produces mature miRNAs (Wei et al., 2024; Wang et al., 2024; Peng et al., 2024a). One strand integrates into the RNA-induced silencing complex (RISC) to silence target mRNAs. MiRNAs play a crucial role in disease processes, including immune responses (Taganov et al., 2006; Peng et al., 2022), cell cycle regulation (Carleton et al., 2007), and tumor invasion (Hussen et al., 2021; Jiang et al., 2024). Abnormal miRNA expression or impairment is linked to various diseases, particularly cancers like breast, pancreatic (Fathi et al., 2021), and lung cancer (Ni et al., 2021). For instance, certain miRNAs are overexpressed in cancer and inhibit the activity of specific tumor suppressor genes, leading to cancer cell proliferation and metastasis. Differential expression of other miRNAs fails to suppress oncogenes, thus making them prime targets for the possible diagnosis and therapeutic interventions in the cancer progression. miRNA-based treatment strategies, such as miRNA mimics and ASOs (Baker et al., 2024), hold significant promise in advancing medical research.

MRNAs, on the other hand, are transcribed from DNA to carry genetic information for protein synthesis in a process called transcription. During this process, RNA polymerase synthesizes mRNA using a DNA template in the nucleus (Qin et al., 2022). The mRNA is subsequently transported to the cytoplasm, where ribosomes translate it into proteins. Beyond protein synthesis, mRNAs play a key role in maintaining cellular homeostasis and facilitating rapid responses to environmental changes (Das et al., 2021), such as stress or nutrient deprivation, by regulating their own synthesis. These dynamic adjustments enable mRNAs to precisely regulate gene expression in changing conditions (Zhao et al., 2021). However, mRNA regulation is not solely intrinsic. Small non-coding RNAs, such as miRNAs, function as sequence-specific inhibitors of mRNA activity, modulating post-transcriptional processes and influencing both mRNA stability and translation efficiency.

The regulation of gene expression by miRNAs and mRNAs is mediated through highly specific and dynamic interactions. A single miRNA can target multiple mRNAs, while a single mRNA may be regulated by multiple miRNAs. In most cases, miRNA binding sites are located in the 3′untranslated region (3′UTR) of mRNAs (Griesemer et al., 2021). In certain instances, miRNA binding sites may also be found within the coding sequence (CDS) or the 5′untranslated region (5′UTR) (Chekulaeva, 2023). The interaction between miRNAs and their target mRNAs results in either translational inhibition or mRNA degradation, thereby modulating specific protein expression levels. Studies indicate that miRNA-mRNA interactions play a critical role in physiological processes such as cell growth, differentiation, and apoptosis, as well as in the initiation and progression of diseases (Laggerbauer and Engelhardt, 2022; Peng et al., 2024b). For instance, aberrant miRNA expression has been implicated in cancer, neurological disorders, and cardiovascular diseases. Understanding miRNA-mRNA interactions provides insight into the underlying molecular mechanisms of these disorders and facilitates the identification of novel therapeutic targets in precision medicine.

Although traditional wet lab methods (e.g., RNA immunoprecipitation, reporter gene assay, and quantitative PCR) have advanced the understanding of miRNA-mRNA interactions, they face several limitations, such as long experimental cycles, high labor costs, and limited scalability for large-scale miRNA-mRNA pair screening. These limitations arise from the inherent characteristics of wet lab methods, rendering them less capable of addressing the intricate regulatory relationships between miRNAs and mRNAs arising in complex biological systems. These challenges have been addressed by machine learning-based computational methods in the study of miRNA-target mRNA interactions. Such approaches have since been integrated with multidimensional biological data that allow high-throughput prediction and analysis. For example, traditional tools, such as TargetScan (McGeary et al., 2019) and miRDB (Chen and Wang, 2020), are based on seed sequence matching and conservation analysis in identifying potential targets, which limits their ability to integrate complex biological features.

Several models like deepTarget (Lee et al., 2016) integrate sequence-based features and nonlinear learning to achieve better model performance. The capabilities of deep learning to process large-scale data have proven successful across various fields. For example, a deep graph convolutional network (DGCN) was developed by Chen et al. to predict the miRNA-disease associations by constructing a unified graph structure, which takes into account potential nonlinear associations among miRNAs and diseases, leading to significant improvements in prediction performance (Zheng et al., 2021; Peng et al., 2021a). Liu et al. proposed the MPCLCDA model for circRNA-disease association prediction, in which heterogeneous networks are created from automatic meta-path selection and contrastive learning, thus effectively predicting circRNA-disease associations using graph convolutional techniques (Liu et al., 2023). Likewise, Guo et al. proposed employing structural deep neural network embedding models to predict circRNA-miRNA interactions, this model combines structural and sequence features to achieve high predictive performance by reconstructing the association matrix (Guo et al., 2022; Peng et al., 2021b). All these works validate significant advantages of deep learning in handling complex biological networks, incorporate multidimensional features, and optimize prediction accuracy, offering methodological insights for miRNA-target mRNA prediction.

Although deep learning has advanced miRNA-target mRNA prediction, current methods have notable limitations in feature integration and attention mechanisms. Existing approaches like miTAR primarily rely on CNN-BiLSTM hybrid architectures to capture spatial and sequential features but lack sophisticated attention mechanisms to dynamically prioritize important features during prediction (Gu et al., 2021). Methods such as miGAP (Yoon et al., 2023) and AEmiGAP (Yoon et al., 2024), while achieving high performance through advanced embedding techniques and autoencoder-based feature extraction, still focus predominantly on sequence-level information without incorporating structural node attributes or topological relationships. These approaches treat miRNA and gene sequences as isolated entities, missing the opportunity to leverage graph-based representations that can capture the inherent network structure of molecular interactions. Furthermore, current methods do not employ attention mechanisms to adaptively weight different feature components, limiting their ability to focus on the most relevant sequence patterns and structural characteristics for specific miRNA-mRNA pairs.

To address these limitations, we propose the GRMMI model, which combines graph structures and attention mechanisms to provide a more comprehensive approach. Unlike existing sequence-only methods, GRMMI integrates both RNA sequence features and node attributes within a unified framework, using CNN-BiLSTM to extract sequence features—CNN captures local patterns and BiLSTM captures extended dependencies. The key innovation lies in the mutual attention mechanism that assigns dynamic importance to features, enabling the model to better capture miRNA-mRNA interactions compared to static feature weighting in current approaches. GRMMI also reverses the mRNA sequence to align with the antisense strand pairing mechanism of miRNAs, enhancing understanding of their complementary binding. Graph embedding techniques represent RNA node features and topological structures, improving the ability of the model to capture RNA interactions beyond pure sequence information. The joint representation of sequence and graph-based features is then used for prediction output.

## Materials and methods

### Data description

**FIGURE 1:**
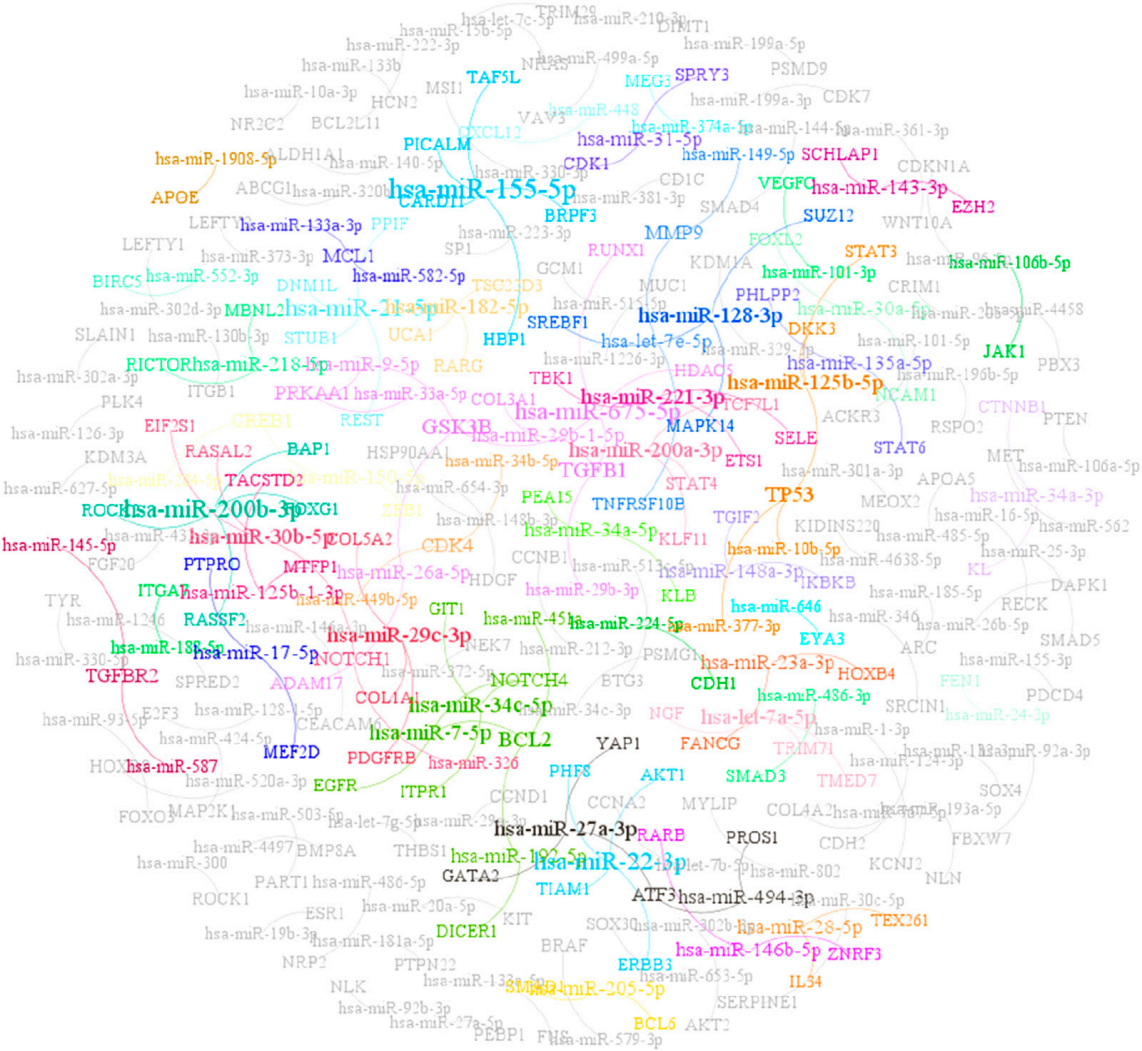
Partial data relationship diagram of MTIS-9214.

MiRTarBase is a widely used bioinformatics database that curates experimentally validated miRNA-mRNA interactions (Huang et al., 2022). It integrates data from experimental techniques like reporter assays, RNA immunoprecipitation, and microarray analyses, ensuring high quality and reliability of miRNA-target gene relationships. From the miRTarBase database, we extracted a dataset of 9,214 experimentally validated miRNA-mRNA interaction pairs, comprising 3,069 mRNAs and 861 miRNAs. We refer to this dataset as MTIS-9214, and the relationships among some of the data are illustrated in Figure 1. The miRNA sequence information was retrieved from miRBase (Kozomara et al., 2019), while the mRNA sequences were obtained from GENCODE-v38 (GRCh38. p14).

To construct a comprehensive dataset for model training and evaluation, we also generated an equal-sized negative sample dataset. The negative samples were created by randomly pairing miRNAs with mRNAs, ensuring that these pairings were not present in the experimentally validated dataset. In other words, these miRNA-mRNA pairs lacked any known experimental evidence. The inclusion of negative samples facilitates the ability of the model to effectively distinguish between true positive interactions and non-functional pairings, which is crucial for reducing false positives and improving predictive accuracy. Ultimately, the size of the negative sample dataset was matched to the positive dataset, with both containing 9,214 interaction pairs. This balanced dataset design eliminates the issue of class imbalance during model training, enhancing the performance of the model and its ability to predict real-world biological interactions.

### Method architecture

**FIGURE 2:**
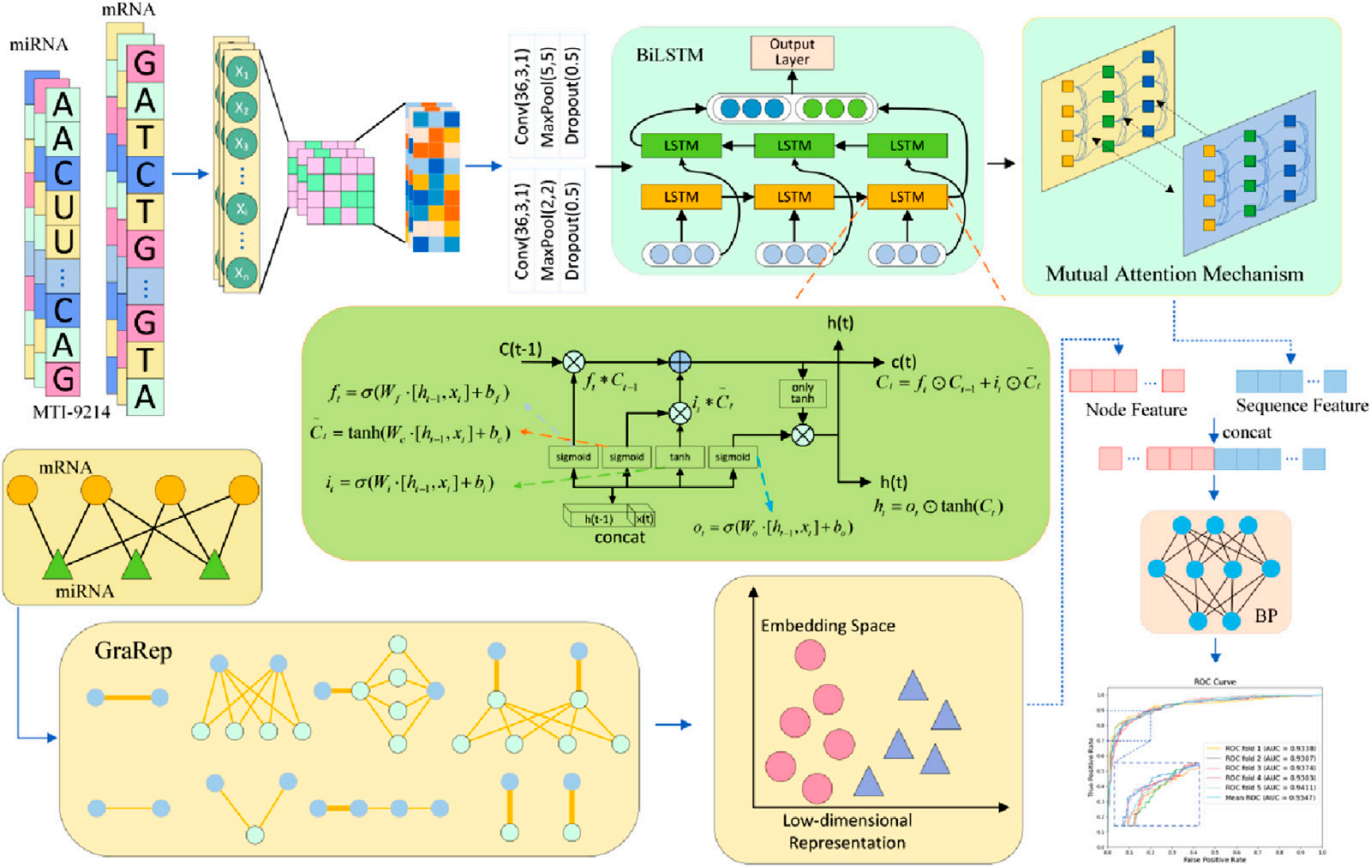
Structural diagram of the GRMMI model.

In this study, we propose a computational framework for GRMMI, aiming to enhance the prediction performance of miRNA-mRNA interaction pairs by extracting and integrating RNA sequence and node features. As illustrated in Figure 2, we first utilize the miRNA sequences from the miRBase database and the mRNA sequences from GRCh38. p14 for pretraining using the FastText model to obtain the initial sequence embedding weights of RNA sequences. Subsequently, the sequence feature extraction module of the GRMMI framework is applied to the data from the MTIS-9214 dataset to extract sequence features. Meanwhile, we employ the GraRep graph embedding method to extract the node features of miRNA-mRNA interaction pairs. Finally, a BP neural network is utilized to fuse the sequence and node features. Based on the extracted features, the model is applied to interaction prediction.

### Pretrained RNA sequence embedding weights extraction

The development of NLP has advanced from rule-based methods to deep learning models. Traditional approaches like bag-of-words (BoW) struggled to capture sequential and semantic relationships. With the introduction of word embedding techniques such as Word2Vec, GloVe (Pennington et al., 2014), and FastText (Joulin et al., 2016), NLP models gained the ability to capture semantic information more effectively. These advancements have been applied to bioinformatics, where RNA sequences, akin to a “language” with nucleotides as “words,” can be analyzed using NLP. Word embeddings represent biological sequences as low-dimensional vectors, capturing complex patterns for prediction and analysis.

**FIGURE 3:**
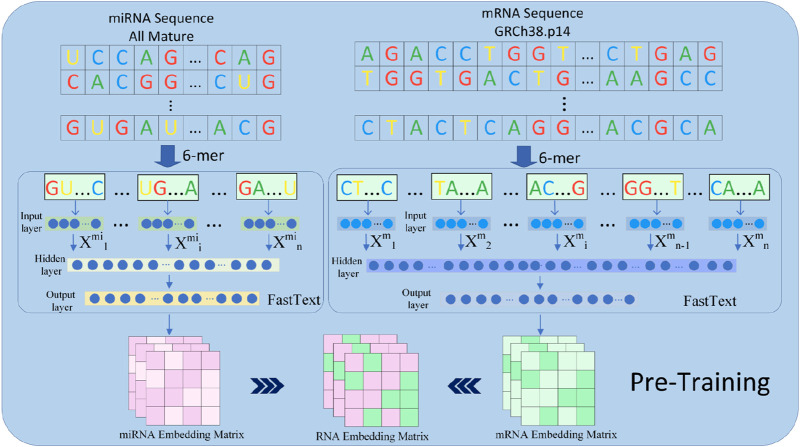
The pretraining component in the GRMMI model. In the process of deriving pre-trained weights for the model, miRNA sequences are utilized in their entirety, whereas mRNA sequences are randomly segmented into fragments ranging from 200 to 400 nucleotides using a sliding window approach. These sequences and fragments are then tokenized into k-mers, which are treated as words. The FastText algorithm is employed to extract sequence weights from the RNA corpus, and these weights are subsequently utilized as the weights for the downstream embedding layer in the model.

In this study, we used FastText as the embedding extraction tool to represent features of miRNA and mRNA sequences. This model leverages subword information of words using subword information. FastText provides word embedding combined with n-gram subword modeling, which makes it suitable for biological sequences with local structural features. The detailed structure and results are shown in Figure 3.

The fundamental idea behind FastText is an extension of the Skip-gram model, representing RNA sequences using n-gram subwords. In the experiment, FastText was employed to pretrain miRNA and mRNA sequences and extract the embedding weights of RNA sequences for downstream embedding layers. Given the relatively long lengths of mRNA sequences, we employed a sliding window approach to segment each mRNA sequence into 200–400 nucleotide fragments with overlapping regions between windows to preserve potential binding site integrity, while miRNA sequences were left unsegmented due to their shorter lengths. Subsequently, each fragment was passed through FastText to generate k-mer embeddings, where k = 6 For an miRNA or mRNA sequence $S=\left\{b_{1},b_{2},...,b_{L}\right\}$ (where $b_{i}$ represents bases A, U, C, and G) of length L the k-mer set $G\left(S\right)$ is defined as shown in Equation 1:

$$G\left(S\right)=\left\{\left.g_{i}\right|g_{i}=b_{i}b_{i+1}...b_{i+5},1\leq i\leq L-5\right\}$$ (1)

where k-mer refers to a sub-sequence of length k = 6 each $g_{i}$ represents a sub-sequence of six bases (e.g., AAAAAA, UUUUG, etc.) Ultimately, a vocabulary κ of k-mers is generated for all mRNA and miRNA sequences, with a size of 4,096 × 64, corresponding to 4^6^ (all possible combinations of the four nucleotide bases A, U, C, and G).

For pretraining k-mer embeddings with FastText, the skip-gram method is employed. The objective is to maximize the co-occurrence probability between the center k-mer and its surrounding k-mers in the sequence. The objective function is defined as shown in Equation 2:

$$J=\sum_{g\in \kappa }\sum_{c\in context\left(g\right)}\log P\left(c\left|g\right.\right)$$ (2)

Let g represent the center k-mer and $context\left(g\right)$ denotes the context k-mers co-occurring with g within a specified window size.

The co-occurrence probability $P\left(c\left|g\right.\right)$ is defined as shown in Equation 3:

$$P\left(c\left|g\right.\right)=\frac{\exp\left({\vec{z_{c}}}^{⊤}\cdot \vec{z_{g}}\right)}{\sum_{k\in \kappa }\exp\left({\vec{z_{c}}}^{⊤}\cdot \vec{z_{g}}\right)}$$ (3)

$\vec{z_{g}}$ represents the embedding vector of the central k-mer, $\vec{z_{c}}$ represents the embedding vector of the contextual k-mer, and κ denotes the set of all k-mers, with a size of 4,096. By optimizing J , FastText is able to learn the embedding vector $\vec{z_{g}}\in R^{64}$ for each k-mer, where the embedding dimension is 64.

### Extraction of RNA sequence embeddings using the GRMMI

Since RNA sequences consist of discrete bases (A, U, C, G/T) as symbolic data, directly inputting them into deep learning models to capture semantic and structural features is challenging. To address this, the GRMMI framework incorporates a sequence feature extraction module designed with an embedding layer, a convolutional neural network (CNN) layer, a bidirectional long short-term memory (BiLSTM) layer, and a mutual attention mechanism layer employing a multi-head attention mechanism. As part of preprocessing, mRNA sequences were reversed from their original 5′to 3′orientation to a 3′to 5′direction, aligning with the antisense strand pairing mechanism of miRNAs. T This adjustment enhances the ability of the model to learn features critical for predicting miRNA-mRNA interactions by simulating complementary base-pairing interactions, ensuring the processed sequence data accurately represents their functional relationships.

Within the GRMMI framework, the sequence feature extraction module first employs an embedding layer to map these discrete symbols into low-dimensional dense vector representations, capturing latent relationships and contextual information between bases. The embedding weights are initialized with pre-trained FastText vectors, leveraging semantic features from large-scale RNA datasets. Next, the model uses a CNN layer to extract local RNA sequence features. This layer identifies significant base patterns for functional inference and, through convolution and pooling, efficiently processes local features, enhances computational efficiency, and reduces data dimensionality. After processing by the CNN layer, the RNA sequence features are represented as shown in Equations 4, 5:

$$H_{\text{mi}}={\text{CNN}}_{\text{mi}}\left(E_{\text{mi}}\right)$$ (4)

$$H_{m}={\text{CNN}}_{m}\left(E_{m}\right)$$ (5)

Here, $E_{\text{mi}}$ and $E_{m}$ represent the embedding representations obtained through the embedding layer, respectively

BiLSTM, an extension of LSTM (Long Short-Term Memory network) (Nguyen et al., 2021), addresses the vanishing and exploding gradient problems in standard RNNs through its gating mechanisms and processes input sequences in both forward and backward directions, enabling it to effectively model long-range dependencies (Aslan et al., 2021). For an input sequence $X=\left\{x_{1},x_{2},...,x_{T}\right\}$ , the state update of an LSTM unit at each time step t is defined by the following equations:

The forget gate $f_{t}$ controls which information from the cell state $C_{t-1}$ of the previous time step is retained and which is discarded, as defined in Equation 6.

$$f_{t}=\sigma \left(W_{f}\cdot \left[h_{t-1},x_{t}\right]+b_{f}\right)$$ (6)

where $W_{f}$ is the weight matrix, $b_{f}$ is the bias vector, σ is the sigmoid activation function, $h_{t-1}$ represents the hidden state from the previous time step, and $x_{t}$ is the input at the current time step.

The input gate $i_{t}$ controls how much new information is written to the cell state at the current time step, as shown in Equation 7.

$$i_{t}=\sigma \left(W_{i}\cdot \left[h_{t-1},x_{t}\right]+b_{i}\right)$$ (7)

The candidate cell state ${\tilde{C}}_{t}$ generates the candidate state for the current time step as given by Equation 8:

$${\tilde{C}}_{t}=\tanh\left(W_{c}\cdot \left[h_{t-1},x_{t}\right]+b_{c}\right)$$ (8)

The cell state update $C_{t}$ combines the forget gate and the input gate to update the current cell state as shown in Equation 9:

$$C_{t}=f_{t}⊙C_{t-1}+i_{t}⊙{\tilde{C}}_{t}$$ (9)

where ⊙ represents element-wise multiplication.

The output gate $o_{t}$ determines which parts of the cell state $C_{t}$ are output, as defined in Equation 10:

$$o_{t}=\sigma \left(W_{o}\cdot \left[h_{t-1},x_{t}\right]+b_{o}\right)$$ (10)

The hidden state $h_{t}$ at the current time step is determined by the output gate and the current cell state according to Equation 11:

$$h_{t}=o_{t}⊙\tanh\left(C_{t}\right)$$ (11)

For a BiLSTM, it consists of two LSTM layers: the forward LSTM processes the sequence $\left\{x_{1},x_{2},...,x_{T}\right\}$ from left to right, while the backward LSTM processes the sequence $\left\{x_{T},x_{T-1},...,x_{1}\right\}$ from right to left. For the input sequence X , the forward and backward LSTM layers compute the hidden states $\vec{h_{t}}$ and $\overset{⃖}{h_{t}}$ , respectively, as shown in Equations 12, 13.

$$\vec{h_{t}}={\text{LSTM}}_{\text{forward}}\left(x_{\text{cnn},t},\vec{h_{t-1}}\right)$$ (12)

$$\overset{⃖}{h_{t}}={\text{LSTM}}_{\text{backward}}\left(x_{\text{cnn},t},\overset{⃖}{h_{t+1}}\right)$$ (13)

The final hidden state $h_{t}$ is obtained by concatenating the forward hidden state and the backward hidden state according to Equation 14:

$$h_{t}=concat\left(\vec{h_{t}},\overset{⃖}{h_{t}}\right)$$ (14)

After being processed by the CNN, the sequence is further modeled by a BiLSTM layer to capture global contextual information. BiLSTM captures both forward and backward dependencies between bases, which is suitable for RNA sequences due to their bidirectional structures and long-range dependencies. This enables a comprehensive representation of the structural and functional features of RNA (Zhang et al., 2022).

The addition of BiLSTM overcomes the limitation of CNN in only capturing local patterns, enhancing feature representation. The sequence data is then processed by a mutual attention mechanism, which captures the interactions between miRNA and mRNA sequences and emphasizes their global dependencies, focusing on the dynamic relationships between the features of both input sequences (Yang et al., 2021).

The miRNA and mRNA feature representations obtained from the BiLSTM layer serve as the inputs to the mutual attention mechanism, as defined in Equations 15, 16:

$$H_{\text{mi}}^{\text{BiLSTM}}=\left\{h_{1}^{\text{mi}},h_{2}^{\text{mi}},...,h_{T}^{\text{mi}}\right\},H_{\text{mi}}\in R^{T\text{mi}\times d}$$ (15)

$$H_{m}^{\text{BiLSTM}}=\left\{h_{1}^{m},h_{2}^{m},...,h_{T}^{m}\right\},H_{m}\in R^{Tm\times d}$$ (16)

Here, Tmi and Tm represent the lengths of the miRNA and mRNA sequences, respectively, and d denotes the feature dimension of the BiLSTM output. These features are used as the inputs for the mutual attention mechanism, serving as Query ( Q ), Key ( K ), and Value ( V ).

The mutual attention mechanism is implemented using multi-head attention. In multi-head attention, each head applies independent weight matrices to linearly transform the input features into Query Q , Key K , and Value V . Specifically, the miRNA features $H_{\text{mi}}^{\text{BiLSTM}}$ are used as Query Q , while the mRNA features $H_{m}^{\text{BiLSTM}}$ are used as Key K and Value V , as shown in Equation 17:

$$\begin{array}{l} Q=H_{\text{mi}}^{\text{BiLSTM}}W_{Q}\\ K=H_{m}^{\text{BiLSTM}}W_{k}\\ V=H_{m}^{\text{BiLSTM}}W_{v} \end{array}$$ (17)

Here, $W_{Q},W_{K},W_{v}\in R^{d\times d_{k}}$ represents the learnable projection weight matrix, and $d_{k}$ is the dimension of a single attention head.

The core of the mutual attention mechanism is to compute the attention scores between miRNA and mRNA sequences to capture their mutual dependencies. Specifically, the similarity score matrix A is first computed through a dot product operation between the Query Q and the Key K . Then, A is normalized to generate attention weights, which are subsequently applied to the Value V to obtain the weighted feature representations. The detailed computation process is as follows:

The similarity score matrix A is calculated as shown in Equation 18:

$$A=\frac{QK^{⊤}}{\sqrt{d_{k}}}$$ (18)

In this formula, $A\in R^{T_{\text{mi}}\times T_{m}}$ represents the similarity between each time step in the miRNA sequence and each time step in the mRNA sequence.

Next, for the similarity score matrix A , the softmax function is applied to normalize each row of the matrix. This operation transforms the unnormalized dot product similarity values into a probability distribution, which quantifies the strength of the correlation between the Query and the Key. The normalized attention weight matrix α is calculated as shown in Equation 19:

$$\alpha_{ij}=\frac{\exp\left(A_{ij}\right)}{\sum_{k=1}^{Tm}\exp\left(A_{ik}\right)},\forall i\in \left[1,T_{\text{mi}}\right],\forall j\in \left[1,T_{m}\right]$$ (19)

Here, $\alpha_{ij}$ represents the attention weight of the i -th time step in the miRNA sequence for the j -th time step in the mRNA sequence. The softmax function ensures that the sum of the weights in each row equals 1, thereby allowing the weight matrix α to probabilistically describe the distribution of attention from different positions in the miRNA sequence to various positions in the mRNA sequence.

Finally, after obtaining the attention weight matrix α , it is used to perform a weighted sum over the value matrix V , generating the final weighted feature representation $H_{\text{attention}}$ . Specifically, for the i -th position in the Query Q , its weighted feature representation $h_{i}^{\text{attention}}$ is computed according to Equation 20:

$$h_{i}^{\text{attention}}=\sum_{j=1}^{T_{m}}a_{ij}v_{j},\forall i\in \left[1,T_{\text{mi}}\right]$$ (20)

Here, $a_{ij}$ represents the normalized attention weight between the i -th time step of the miRNA sequence and the j -th time step of the mRNA sequence. $v_{j}$ is the feature representation of the j -th time step in the value matrix V , and $h_{i}^{\text{attention}}\in R^{d_{k}}$ represents the weighted feature vector. The matrix-form calculation is expressed as shown in Equation 21:

$$H_{\text{attention}}=\alpha V$$ (21)

where $H_{\text{attention}}\in R^{T_{\text{mi}}\times d_{k}}$ is the feature matrix output by the attention mechanism, describing the weighted feature representation of each time step in the miRNA sequence after integrating information from the mRNA sequence.

Through the above operations, the attention mechanism dynamically aggregates key-value information related to the query, thereby generating feature representations that effectively capture the interaction relationships between miRNA and mRNA sequences (Wang et al., 2023).

### RNA node feature extraction

Since miRNA and mRNA are distinct nodes with complex functional associations, and their interaction relationships are sparse (each miRNA is associated with only a limited number of mRNAs), miRNA can indirectly influence other miRNAs through multiple steps. To capture RNA node features, we use the GraRep method, which embeds graph nodes into a low-dimensional vector space while preserving structural information. By applying matrix factorization, GraRep captures both local and global relationships between nodes, revealing multi-order adjacency relationships (Cao et al., 2015). The first-order adjacency matrix captures direct miRNA-mRNA relationships, while higher-order matrices uncover indirect ones. The embeddings of GraRep provide a unified feature space for miRNA and mRNA, reflecting their complex interactions. The process is detailed in Table 1.

**TABLE 1:** GraRep Algorithm for miRNA-mRNA Node Embedding.

Algorithm 1: GraRep Algorithm for miRNA-mRNA Node Embedding
**Input:** Adjacency matrix $S\in R^{N\times N}$ of miRNA-mRNA interaction graph Maximum transition step K **,** Log shifted factor β Dimension of representation vector d **Output:** Matrix of the miRNA-mRNA graph representation $W\in R^{N\times \left(K\cdot d\right)}$ /* Step 1: Get k -step transition probability matrix */ 1: Compute the transition probability matrix $A=D^{-1}S$ , where D the diagonal degree matrix of S 2: Calculate matrix powers $A^{1},A^{2},...A^{K}$ /* Step 2: Get each K -step representations */ 3: **for** k = 1 to K **do** /* Step 2.1: Get positive log probability matrix */ 4: **for** $j\in \left[1,N\right]$ **do** 5: $\Gamma_{j}^{k}=\sum_{p}A_{p,j}^{k}$ 6: $X_{i,j}^{k}=\log\left(A_{i,j}^{k}+\varepsilon \right)-\log\left(\beta \right)$ 7: Assign negative entries of $X^{k}$ to 0 /* Step 2.2: Construct the representation vector $W^{k}$ */ 8: Perform SVD: $\left[U^{k},\sum^{k},{\left(V^{k}\right)}^{T}\right]=\text{SVD}\left(X^{k}\right)$ 9: $W^{k}=U_{d}^{k}{\left(\sum_{d}^{k}\right)}^{\frac{1}{2}}$ /* Step 3: Concatenate all k -step representation */ 10: $W=\left[W^{1},W^{2},...,W^{K}\right]$ 11: return W

#### Step-1 Get k -step transition probability matrix $A^{k}$

First, the adjacency matrix S is constructed. The input graph is the miRNA-mRNA interaction graph, where the nodes V are composed of miRNA and mRNA, and the edges E represent the known interactions between miRNA and mRNA pairs. Next, the adjacency matrix is normalized. The degree matrix D of the nodes is computed, and S is degree-normalized to obtain the probability transition matrix A . Finally, the k -order adjacency probability matrix $A^{K}$ is computed recursively.

The matrix $A^{K}$ captures the global interaction relationships between miRNA and mRNA within a k -step range. It represents the probability of miRNA or mRNA indirectly influencing other nodes through intermediate nodes.

#### Step-2 Get each k -step representations

The embeddings $W^{k}$ are generated step by step. First, the row normalization factor of the k -order probability matrix is calculated, where $\Gamma_{j}^{k}$ represents the normalized weight of node j under the k -order adjacency relationship. Next, a logarithmic transformation is applied to $A^{k}$ , yielding the k -order logarithm-transformed matrix $X^{k}$ . Here, ε is added to prevent numerical instability caused by log (0), and β is the logarithmic smoothing factor, which enhances significant interaction relationships while reducing noise. Afterward, negative values in $X^{k}$ are set to 0.

Singular value decomposition (SVD) is then performed on $X^{k}$ , and the top d singular vectors are extracted to generate the k -order embedding $W^{k}$ .

#### Step-3 Concatenate all k -step representations

By concatenating the embeddings from all orders, the final node feature matrix W is generated, where $W\in R^{\left|V\right|\times \left(K\cdot d\right)}$ represents the final node embeddings for miRNA and mRNA. These embeddings encapsulate multi-order relational information, ranging from local to global relationships.

## Experiments and results

### Evaluation criteria

In the experiments, the model performance was evaluated using the following eight metrics: Accuracy (Acc), F1-score (F1), Area Under the ROC Curve (AUC), Area Under the Precision-Recall Curve (AUPR), Matthews Correlation Coefficient (MCC), Sensitivity (SEN), Precision (PPV), and Specificity (TNR). The formulas for these metrics are shown in Equations 22–27:

$$\text{ACC}=\frac{\text{TP}+\text{TN}}{\text{TP}+\text{TN}+\text{FP}+\text{FN}}$$ (22)

$$F1=2\cdot \frac{\text{PPV}\cdot \text{Recall}}{\text{PPV}+\text{Recall}}$$ (23)

$$\text{MCC}=\frac{\text{TP}\cdot \text{TN}-\text{FP}\cdot \text{FN}}{\sqrt{\left(\text{TP}+\text{FP}\right)\left(\text{TP}+\text{FN}\right)\left(\text{TN}+\text{FP}\right)}\left(\text{TN}+\text{FN}\right)}$$ (24)

$$\text{SEN}\left(\text{Recall}/\text{TPR}\right)=\frac{\text{TP}}{\text{TP}+\text{FN}}$$ (25)

$$\text{PPV}=\frac{\text{TP}}{\text{TP}+\text{FP}}$$ (26)

$$\text{TNR}=\frac{\text{TN}}{\text{TN}+\text{FP}}$$ (27)

Here, TP (True Positives) refers to the number of samples correctly predicted as positive, TN (True Negatives) refers to the number of samples correctly predicted as negative, FP (False Positives) refers to the number of samples incorrectly predicted as positive, and FN (False Negatives) refers to the number of samples incorrectly predicted as negative.

## Results

**FIGURE 4:**
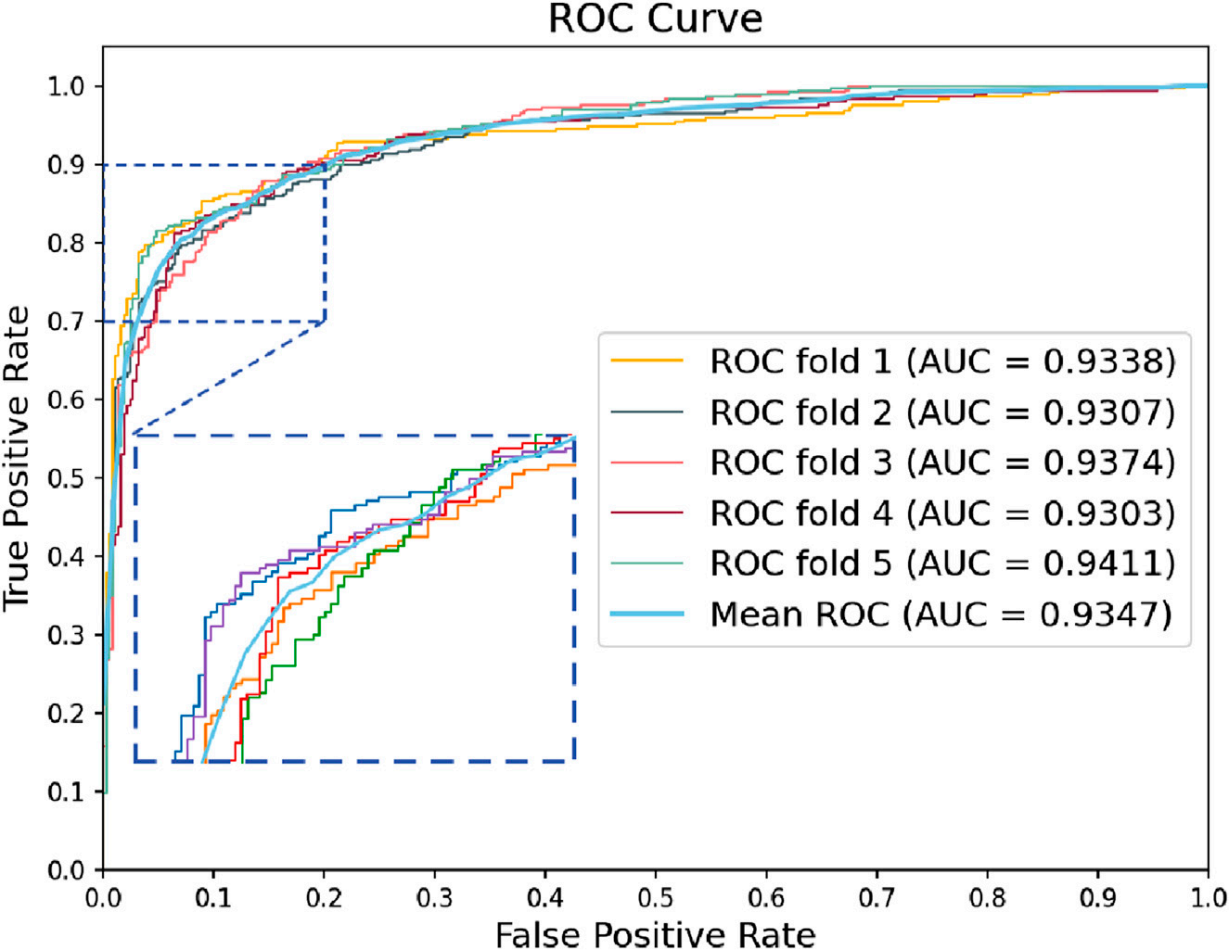
Roc curves of the GRMMI model from 5-fold cross-validation.

We used GRMMI to perform 5-fold cross-validation on the MTIS-9214 dataset to comprehensively evaluate the performance of the model. Additionally, we replaced different components of the model to analyze their impact on overall performance, thereby investigating the contribution of each component to the specific task. Specifically, we replaced the BiLSTM, attention mechanism, feature extraction module, and fusion strategy with other commonly used methods for comparative experiments. Performance changes were analyzed using metrics such as ACC and F1-score calculated with a fixed classification threshold of 0.5 to validate the advantages and robustness of GRMMI in predicting miRNA-mRNA interaction pairs. The detailed results for each fold are shown in Table 2 and Figure 4.

**TABLE 2:** Cross-validation results of GRMMI model using 5-fold testing.

Test set	ACC (%)	F1 (%)	AUC	AUPR	MCC (%)	SEN (%)	PPV (%)	TNR (%)
1	87.67	87.17	0.9338	0.9471	75.57	83.74	90.88	91.60
2	85.35	85.12	0.9307	0.9396	70.73	83.74	86.55	86.96
3	85.89	85.71	0.9374	0.9393	71.80	84.55	86.91	87.23
4	86.43	86.07	0.9303	0.9368	72.95	83.97	88.29	88.89
5	87.92	87.27	0.9411	0.9431	76.23	82.88	92.15	92.95
average	86.65	86.27	0.9347	0.9412	73.46	83.78	88.96	89.53
Std	1.11	0.93	0.0046	0.0040	2.38	0.60	2.47	2.66

## Comparison of different pretraining methods

In the GRMMI model, we used FastText for RNA pretraining to obtain sequence weights suitable for the embedding layer in downstream tasks. To verify the effectiveness of FastText in RNA sequence feature pretraining, we conducted a comparative experiment where Word2Vec was used as an alternative pretraining method. The RNA sequences were pretrained using Word2Vec, and the resulting embedding weights were applied to the GRMMI model for performance evaluation.

**FIGURE 5:**
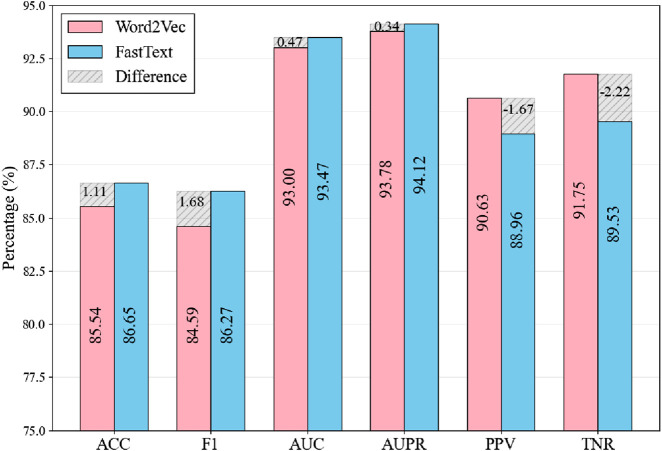
Comparison of Results Using Different Word Embedding Methods. The shaded regions indicate the metric differences between FastText and Word2Vec, calculated as FastText values minus Word2Vec values.

Figure 5 compares Word2Vec and FastText across six performance metrics, with pink bars representing Word2Vec and blue bars representing FastText. The results show mixed performance between the two models across different evaluation metrics. FastText achieves higher scores in Accuracy (86.65% vs. 85.31%), F1 Score (86.27% vs. 84.59%), and AUC (93.06% vs. 92.47%). However, the differences are relatively modest, with AUPR showing only a marginal advantage for FastText (94.12% vs. 93.78%). Conversely, Word2Vec demonstrates better performance in PPV (91.75% vs. 88.96%) and TNR (89.53% vs. 88.96%). The shaded regions highlight these performance differences between the two models. While the ability of FastText to decompose words into subwords enables it to capture character-level features and local patterns in RNA sequences, the overall performance comparison suggests that FastText is more suitable for this study, as it shows advantages in key comprehensive metrics that are important for the overall model evaluation.

### Sequence feature extraction using different deep learning frameworks

For RNA sequence embedding extraction, the GRMMI framework employs a sequence feature extraction module designed with an embedding layer, followed by CNN, BiLSTM, and mutual attention mechanisms. To analyze the contribution of the mutual attention mechanism and BiLSTM, we conducted ablation experiments while keeping the embedding extraction and fusion methods unchanged. GRMMI-noatt removes the mutual attention mechanism, retaining CNN and BiLSTM. GRMMI-lstm replaces BiLSTM with a unidirectional LSTM. GRMMI-nolstm removes the BiLSTM layer entirely, using CNN and the mutual attention mechanism after the embedding layer.

Through the results in Table 3, we see that removing the mutual attention mechanism causes the biggest drop in performance, especially in F1 and MCC, highlighting its role in capturing dependencies between sequences. Replacing BiLSTM with unidirectional LSTM reduces performance but remains better than having no LSTM, demonstrating the importance of BiLSTM for context and sequence modeling. Overall, modular synergies ensure strong RNA sequence feature extraction by integrating various facets of GRMMI.

**TABLE 3:** 5-Fold cross-validation results of ablation study.

Method	ACC (%)	F1 (%)	AUC	AUPR	MCC (%)	SEN (%)	PPV (%)	TNR (%)
noatt	83.32	82.82	0.9132	0.9133	66.78	80.41	85.44	86.21
lstm	84.45	84.73	0.9206	0.9240	68.97	**86.22**	83.31	82.69
nolstm	83.91	83.36	0.9139	0.9161	68.05	80.52	86.50	87.30
GRMMI	**86.65**	**86.27**	**0.9347**	**0.9412**	**73.46**	83.78	**88.96**	**89.53**

Values in bold represent the maximum values for each evaluation metric.

### Feature extraction using different graph embedding methods

For node feature extraction, we observed favorable results using the GraRep graph embedding method. To evaluate the performance of other mainstream methods, we conducted a comparative experiment using various graph embedding approaches, including LINE, DeepWalk, Graph Factorization (GF), HOPE, and Laplacian Eigenmaps (LAP).

Through the results in Table 4, we see that GRMMI achieves the best performance across nearly all metrics, followed by Lap, while DeepWalk demonstrates the worst performance. This may be attributed to the reliance of DeepWalk on the random walk mechanism, which primarily captures local node relationships. However, the miRNA-mRNA network exhibits a complex global topology that cannot be adequately represented by only considering local information. The performance of GRMMI can be attributed to its use of the GraRep method, which captures the structural characteristics and higher-order relationships within the miRNA-mRNA network. This result supports the applicability of the GraRep method in modeling complex miRNA-mRNA networks.

**TABLE 4:** 5-Fold cross-validation results of different graph embedding methods.

Method	ACC (%)	F1 (%)	AUC	AUPR	MCC (%)	SEN (%)	PPV (%)	TNR (%)
Line	78.02	78.17	0.8496	0.8609	56.06	78.68	77.69	77.37
DeepWalk	59.63	38.85	0.7253	0.7150	26.14	25.72	79.75	**93.54**
Gf	70.02	66.27	0.7777	0.7843	41.08	58.93	75.75	81.12
Hope	80.28	80.19	0.8742	0.8804	60.56	79.82	80.56	80.74
Lap	83.99	83.59	0.9149	0.9099	68.09	81.45	85.85	86.54
GRMMI	**86.65**	**86.27**	**0.9347**	**0.9412**	**73.46**	**83.78**	**88.96**	89.53

Values in bold represent the maximum values for each evaluation metric.

### Comparison of different embedding dimensions

In the GRMMI method, the embedding dimension for both sequence features and node features was set to 64. To verify whether 64 is the optimal embedding dimension, we conducted a comparative experiment by varying the embedding dimensions. The dimensions were divided into eight intervals: 16, 32, 48, 64, 72, 96, 112, and 128.

**FIGURE 6:**
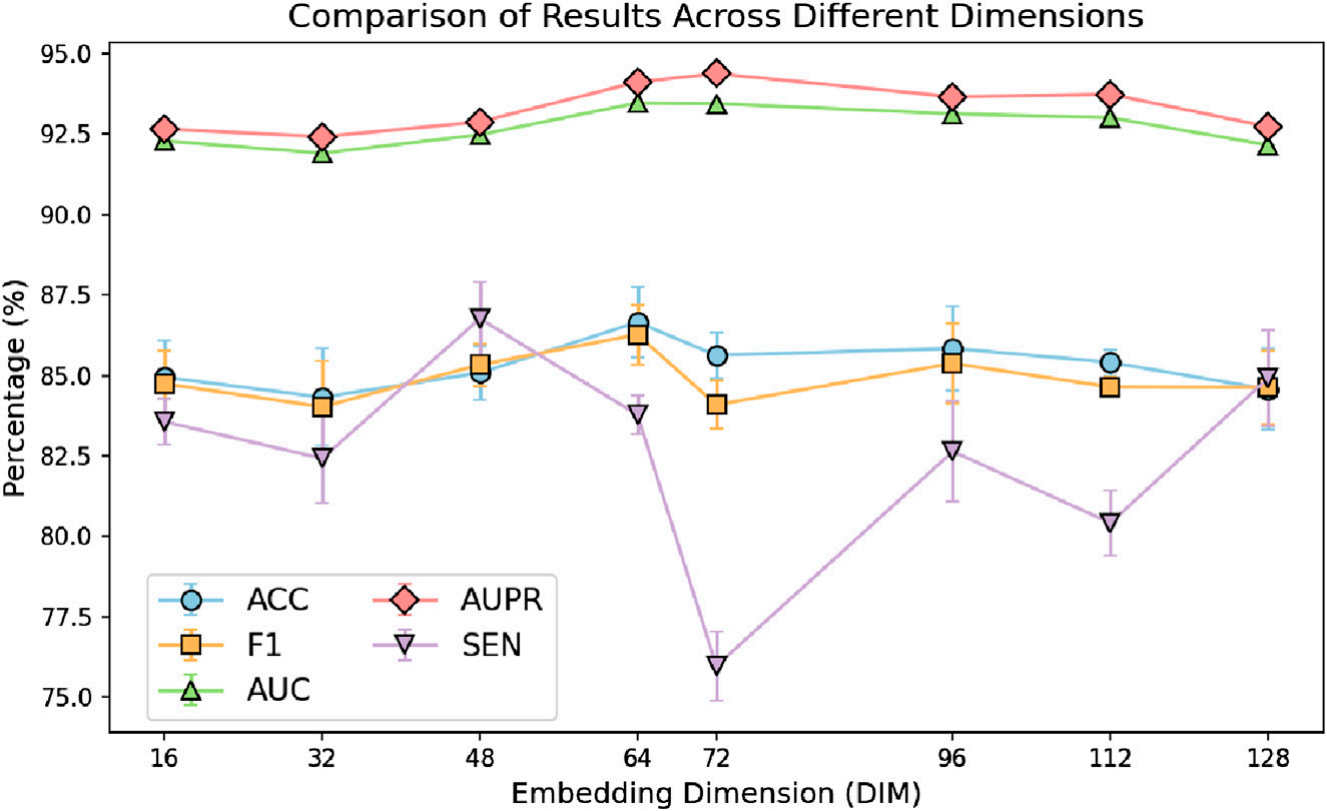
Comparison of results across different dimensions.

From the analysis of the results in Figure 6, it can be observed that changes in embedding dimensions significantly affect the performance of the model. As the embedding dimension increases, the model demonstrates improvement in certain metrics; however, after surpassing 64 dimensions, the performance gains begin to diminish slightly. This could be attributed to the fact that lower-dimensional embeddings are insufficient to fully represent the complex miRNA-mRNA network structure. On the other hand, excessively high dimensions may introduce redundant information, leading to increased noise, which ultimately affects the generalization ability of the model.

### Comparison of different fusion methods

**FIGURE 7:**
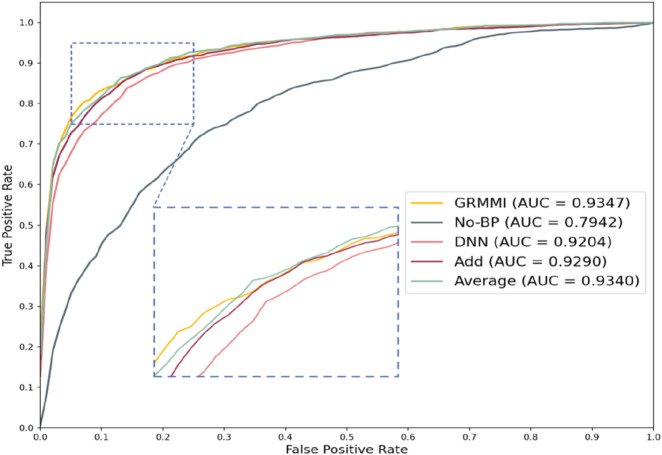
Roc curves of different fusion strategies.

To evaluate the impact of different feature fusion methods on predicting miRNA-mRNA interactions, ablation experiments were designed focusing solely on the feature fusion stage while keeping the sequence and node feature extraction methods unchanged. The GRMMI method fuses sequence and node features using the Concatenate method, followed by nonlinear processing. Variations tested include replacing the Concatenate method with additive or average fusion, using a deep neural network (DNN) instead of the original fusion method while retaining Concatenate, and bypassing the additional processing layer entirely by directly inputting the features into the prediction layer after Concatenate. Table 5 presents the evaluation metrics of GRMMI and its variant models under 5-fold cross-validation, showing the average results across folds with the highest values for each metric highlighted in bold. The ROC curves for different fusion methods are illustrated in Figure 7.

**TABLE 5:** Results of different fusion strategies.

Method	ACC (%)	F1 (%)	AUC	AUPR	MCC (%)	SEN (%)	PPV (%)	TNR (%)
Add	85.84	85.16	0.9291	0.9355	72.01	81.23	**89.53**	**90.45**
Average	86.16	85.63	0.9343	0.9398	72.57	82.42	89.15	89.91
DNN	84.32	83.93	0.9204	0.9241	68.76	81.82	86.2	86.81
No-BP	70.92	67.17	0.7942	0.7786	43.01	59.47	77.22	82.36
GRMMI	**86.65**	**86.27**	**0.9347**	**0.9412**	**73.46**	**83.78**	88.96	89.53

Values in bold represent the maximum values for each evaluation metric.

The results show that the No-BP method, which directly inputs concatenated features into the prediction layer without nonlinear processing, performs significantly worse than methods with nonlinear processing (e.g., BP or DNN). This highlights the importance of nonlinear transformations in capturing complex feature relationships. Nonlinear processing is critical for miRNA-mRNA interaction prediction, as it captures feature interdependencies, while the No-BP method fails to express these patterns, leading to a decline in metrics like AUC and F1-score. The BP method demonstrates stable performance by efficiently mining fused features through fully connected layers and activation functions. Although DNN theoretically captures more complex patterns, it underperforms due to the current data scale, suggesting that moderately complex models like BP are more advantageous for tasks with complex feature relationships, while overly complex models like DNN may be limited by data volume or computational resources.

### Comparison of different classifiers

To evaluate the classification performance of different classifiers on fused features, we designed a comparative experiment using various classifiers. In the original experiment, a fully connected layer was used to output classification results. To further investigate, we introduced multiple traditional machine learning classifiers for comparison and evaluated their performance.

**FIGURE 8:**
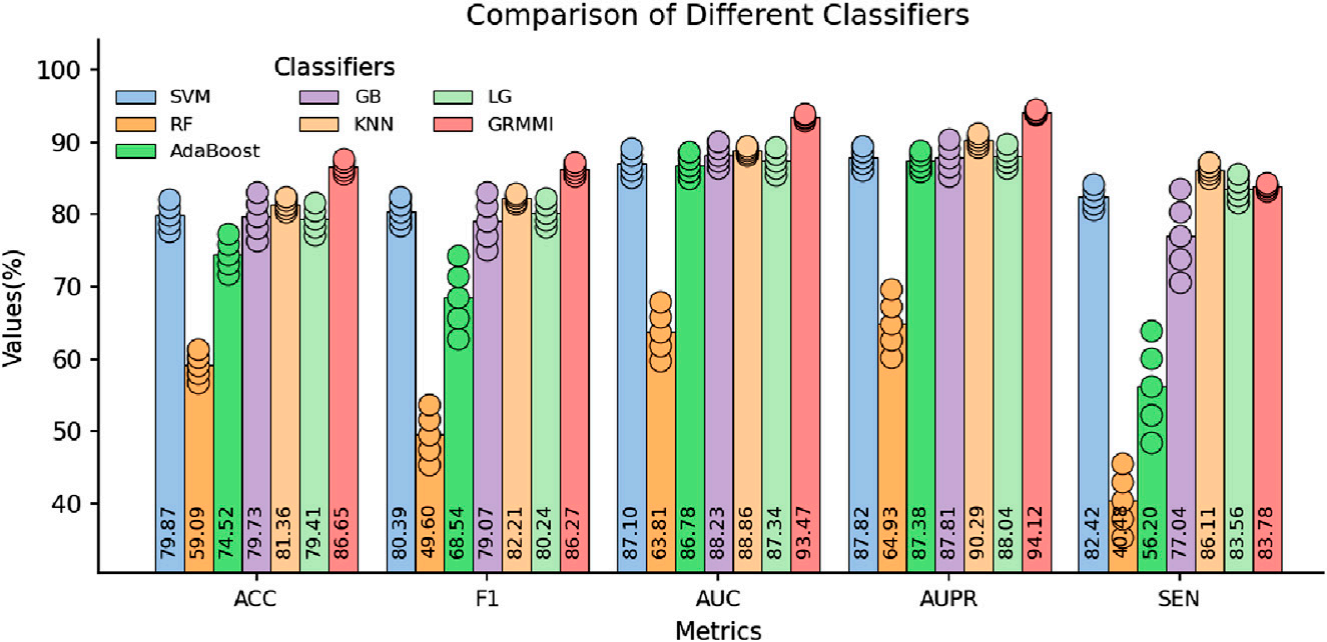
Comparison of Selected Metrics Across Different Classifiers. The circles represent the standard deviation range from 5-fold cross-validation, indicating performance variability for each classifier.

In the experiments, other settings were kept unchanged, and the fused features from the BP neural network were used as input. Six traditional machine learning classifiers were tested, including SVM, Random Forest, AdaBoost, Gradient Boosting, KNN, and Logistic Regression. The results were evaluated using 5-fold cross-validation and are presented in Figure 8.

The results show that while traditional classifiers can perform classification, their performance, especially in metrics like AUC, AUPR, and MCC, is inferior to GRMMI. GRMMI achieves an MCC of 73.46%, outperforming KNN (63.03%) and other classifiers, most of which fall below 60%. Notably, Random Forest performs poorly, with an MCC of only 19.7%. These findings highlight the ability of GRMMI to deliver balanced and consistent results, capturing complex nonlinear feature relationships that traditional classifiers struggle to model.

### Comparison with mainstream prediction algorithms

We compared GRMMI with several mainstream miRNA-mRNA prediction algorithms and state-of-the-art deep learning methods. The traditional algorithms include PITA (based on binding energy changes and target site accessibility) (Kertesz et al., 2007), miRmap (integrating multiple parameters to evaluate miRNA-mediated gene repression) (Vejnar and Zdobnov, 2012), microT (based on seed region matching rules combined with conservation information) (Maragkakis et al., 2009), miRanda (combining base pairing and binding energy calculation for initial screening) (Betel et al., 2010), and PicTar (leveraging multi-species conservation for joint prediction) (Chen and Rajewsky, 2006). Additionally, we compared against three representative deep learning baseline methods: miTAR, a hybrid deep learning approach that integrates CNN and bidirectional RNN layers to learn both spatial and sequential features from raw miRNA and target sequences (Gu et al., 2021). miGAP, a deep learning method that leverages protein2Vec embedding and LSTM-based architecture for miRNA-gene association prediction (Yoon et al., 2023); and AEmiGAP, an advanced deep learning model that integrates autoencoders with LSTM networks to capture latent relationships between miRNAs and genes (Yoon et al., 2024).

Through the results in Table 6, GRMMI shows improved performance compared to both traditional miRNA-mRNA prediction algorithms and other deep learning methods across evaluated metrics. Compared to traditional methods, this performance can be attributed to the integration by GRMMI of diverse RNA sequence and structural features combined with deep learning techniques, which enables more comprehensive capture of the complex regulatory relationships between miRNAs and mRNAs. The nonlinear fitting capability of deep learning models allows GRMMI to extract latent biological information from high-dimensional features while reducing the reliance on specific rules, such as seed region matching or evolutionary conservation, that is common in traditional algorithms. When compared to other deep learning approaches, GRMMI demonstrates advantages in capturing both local and global sequence patterns through its graph-based architecture.

**TABLE 6:** Evaluation of mainstream prediction algorithms and GRMMI.

Method	ACC (%)	F1 (%)	AUC	AUPR	SEN (%)
PITA	61.45	48.55	0.6565	0.8402	32.31
miRmap	57.75	40.73	0.6235	0.8221	25.79
microT	58.19	41.68	0.6276	0.8250	26.53
miRanda	58.30	41.92	0.6286	0.8255	26.73
PicTar	56.94	38.52	0.6169	0.8248	23.96
miTAR	80.46	81.42	0.9027	0.9006	76.51
miGAP	83.12	82.99	0.9056	0.9105	**83.78**
AEmiGAP	81.97	83.15	0.9164	0.9191	76.56
GRMMI	**86.65**	**86.27**	**0.9349**	**0.9412**	**83.78**

Values in bold represent the maximum values for each evaluation metric.

### Case studies

In this section, we performed case studies to demonstrate the capability of the GRMMI model in predicting potential miRNA-mRNA interaction pairs. All experimentally validated miRNA-mRNA pairs were used to train the GRMMI model, which was then applied to predict unknown interactions. The model assigned probability scores to each unknown interaction, ranked in descending order. The top 20 predicted interactions were selected for further analysis and inputted for queries in the miRWalk database.

The results of the miRWalk queries are shown in the table below (Dweep and Gretz, 2015). In this table: PubMed indicates interactions that have been experimentally validated and documented in PubMed. TargetScan indicates interactions that were also predicted by the TargetScan tool. miRDB indicates interactions that were predicted by the miRDB database. Unconfirmed indicates interactions that have not yet been experimentally validated or predicted by any of these tools. The detailed results are as shown in Table 7.

**TABLE 7:** Validation of the top 20 predicted interaction pairs by GRMMI.

Rank	miRNA	mRNA	Evidence
1	hsa-miR-29a-3p	CACNA1C	PubMed TargetScan miRDB
2	hsa-miR-429	AKT2	TargetScan
3	hsa-miR-204-5p	RUNX2	PubMed TargetScan miRDB
4	hsa-miR-206	AGO1	TargetScan
5	hsa-miR-302a-3p	SIRT1	PubMed TargetScan miRDB
6	hsa-miR-181c-5p	PTEN	TargetScan PubMed
7	hsa-miR-22-3p	PPARA	PubMed
8	hsa-miR-27b-3p	FOXO1	PubMed TargetScan miRDB
9	hsa-miR-34a-5p	CDK6	PubMed TargetScan miRDB
10	hsa-miR-324-3p	MYC	PubMed
11	hsa-miR-200a-3p	LPP	PubMed TargetScan miRDB
12	hsa-miR-141-5p	VEGFA	TargetScan miRDB
13	hsa-miR-548b-3p	IRS1	miRDB
14	hsa-miR-212-5p	RECK	miRDB
15	hsa-miR-122-5p	WNT1	PubMed
16	hsa-miR-921	BCL2	**Unconfirmed**
17	hsa-miR-451a	TTN	TargetScan miRDB
18	hsa-miR-873-5p	ZNF805	TargetScan miRDB
19	hsa-miR-106b-5p	ESR1	TargetScan miRDB
20	hsa-miR-518b	KRAS	**Unconfirmed**

The relationships between miRNA and mRNA are crucial for understanding disease mechanisms and developing therapeutic strategies. Exploring these interactions can provide new insights and potential targets for molecular diagnostics, drug development, and precision medicine.

For example, hsa-miR-29a-3p targets CACNA1C, playing a key role in atrial fibrillation by disrupting calcium homeostasis in cardiomyocytes. This discovery offers a new target for intervention (Zhao et al., 2016). Similarly, hsa-miR-204-5p targets RUNX2, regulating vascular smooth muscle cell calcification. By inhibiting RUNX2 expression, it reduces vascular calcification (Cui et al., 2012), providing a basis for developing miRNA-based therapies for atherosclerosis and cardiovascular diseases.

## Conclusion

The identification of miRNA-mRNA interactions is crucial for understanding disease mechanisms and developing therapeutic interventions. These regulatory relationships significantly influence gene expression and play essential roles in the onset and progression of various diseases, including cancer and cardiovascular disorders. This study proposed an innovative deep learning model, GRMMI, to enhance the accuracy of miRNA-mRNA interaction predictions.

The GRMMI model integrates sequence features and node features using a CNN-BiLSTM architecture combined with mutual attention mechanisms and graph embedding techniques. During data preprocessing, to better reflect the complementary pairing mechanism between miRNA and mRNA, the mRNA sequences were reversed from their original 3′to 5′orientation to a 5′to 3′orientation. This adjustment aligns the direction of mRNA sequences with that of miRNA, enabling the model to more accurately learn the pairing relationships and regulatory effects between the two. By effectively fusing these features through a backpropagation neural network, the model demonstrates significant advantages in predictive performance compared to traditional and alternative methods. Case studies further validated the effectiveness of the GRMMI model in identifying potential miRNA-mRNA interactions and demonstrated its practical significance in biological research.

Despite its promising predictive performance, the GRMMI model has some limitations. For example, there is room for improvement in the integration of multi-dimensional features, and its adaptability to larger-scale real-world biological datasets requires further validation. Additionally, the complexity of the model may impose higher computational resource requirements, which could be a limitation for large-scale applications. Future research will focus on optimizing feature extraction and fusion strategies to reduce the introduction of redundant information, improving the adaptability of the model to sparse data, and integrating more biological information into the model.

## Data Availability

The original contributions presented in the study are included in the article/supplementary material, further inquiries can be directed to the corresponding authors.
